# Supplementary material for: Ferroptosis Inducers Upregulate PD-L1 in Recurrent Triple-Negative Breast Cancer
Source: Cancers (Basel). 2023 Dec 28;16(1):155. doi: 10.3390/cancers16010155 (PMC10778345; doi:10.3390/cancers16010155)
Supplement: Supplementary file 1 [file cancers-16-00155-s001.zip › cancers-2767291-supplementary.pdf]

cancers

Article

Ferroptosis Inducers Upregulate PD-L1 in Recurrent Triple-Negative Breast Cancer

Christophe Desterke [1,*], Yao Xiang [2], Rima Elhage [2,3], Clémence Duruel [2,3], Yunhua Chang [2,*] and Ahmed Hamaï [2,3,*]

1 UFR Médecine-INSERM UMRS1310, Université Paris-Saclay, F-94800 Villejuif, France
2 INSERM UMR-S1151, CNRS UMR-S8253, Institut Necker Enfants Malades, Université Paris Cité, F-75015 Paris, France; yao.xiang@inserm.fr(Y.X.); rima.elhage@inserm.fr (R.E.); clemence.duruel@inserm.fr (C.Du.)
3 Team 5/Ferostem Group, F-75015 Paris, France
* Correspondence: christophe.desterke@inserm.fr (C.D.); ahmed.hamai@inserm.fr (A.H.)

SUPPLEMENTAL FIGURES

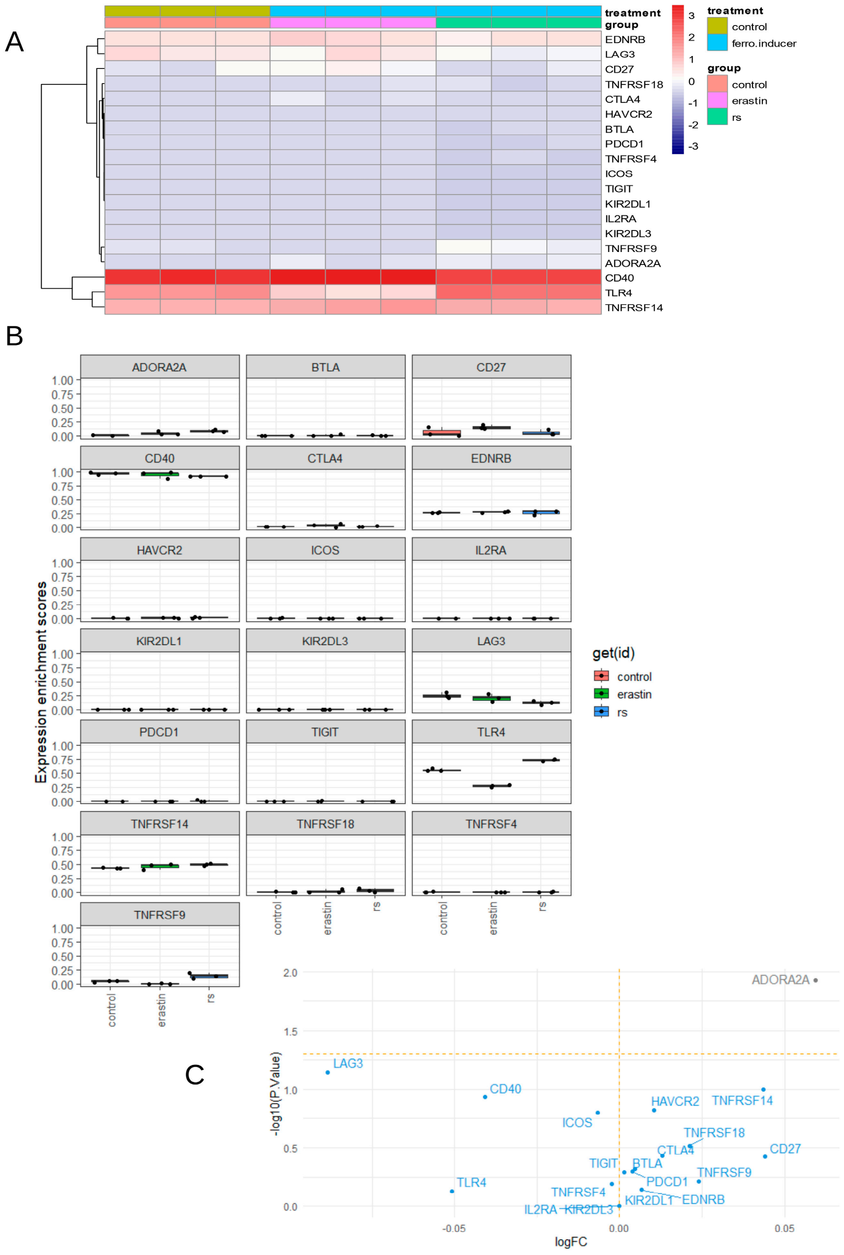

Figure S1. Regulation of immune receptor in MDA-MB-231 after treatment with ferroptosis inducers (erastin and RSL3): (A) Expression heatmap of immune receptors according experimental conditions: untreated control, erastin, RS (RSL3); (B) Expression boxplots of immune receptors; (C) Volcanoplot of differential expression gene analysis for immune receptors between control versus ferroptosis inducer treatments.

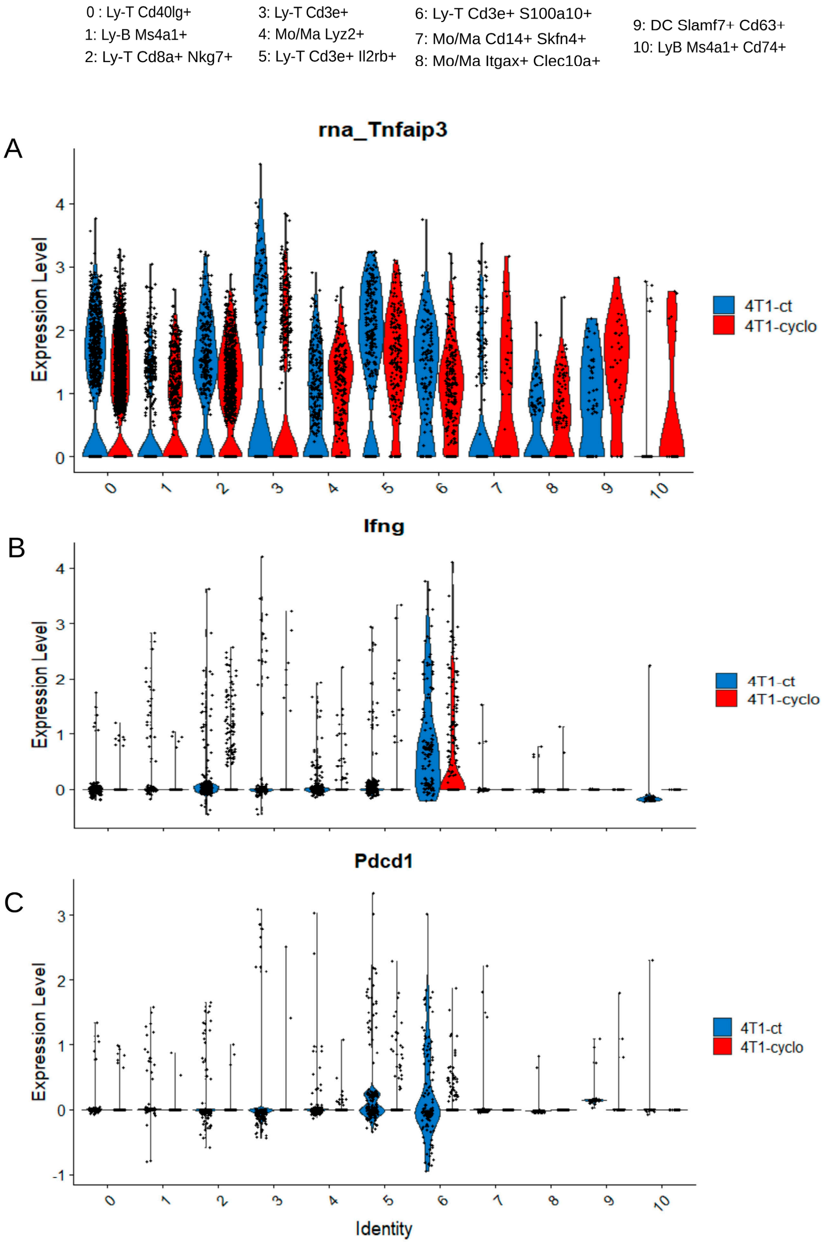

Figure S2. Cyclophosphamide dependent regulation of single cell expression for Tnfaip3, Ifng, Pdcd1 in immune micro-environment of 4T1 transplanted tumors: (dataset GSE191246): CT: untreated tumor, cyclo: tumor with cyclophosphamide treatment (ferroptosis inducers) (A) Volcanoplot of Tnfaip3 single cell expression stratified by cell clusters; (B) Volcanoplot of Ifng single cell expression stratified by cell clusters; (C) Volcanoplot of Pdcd1 single cell expression stratified by cell clusters.

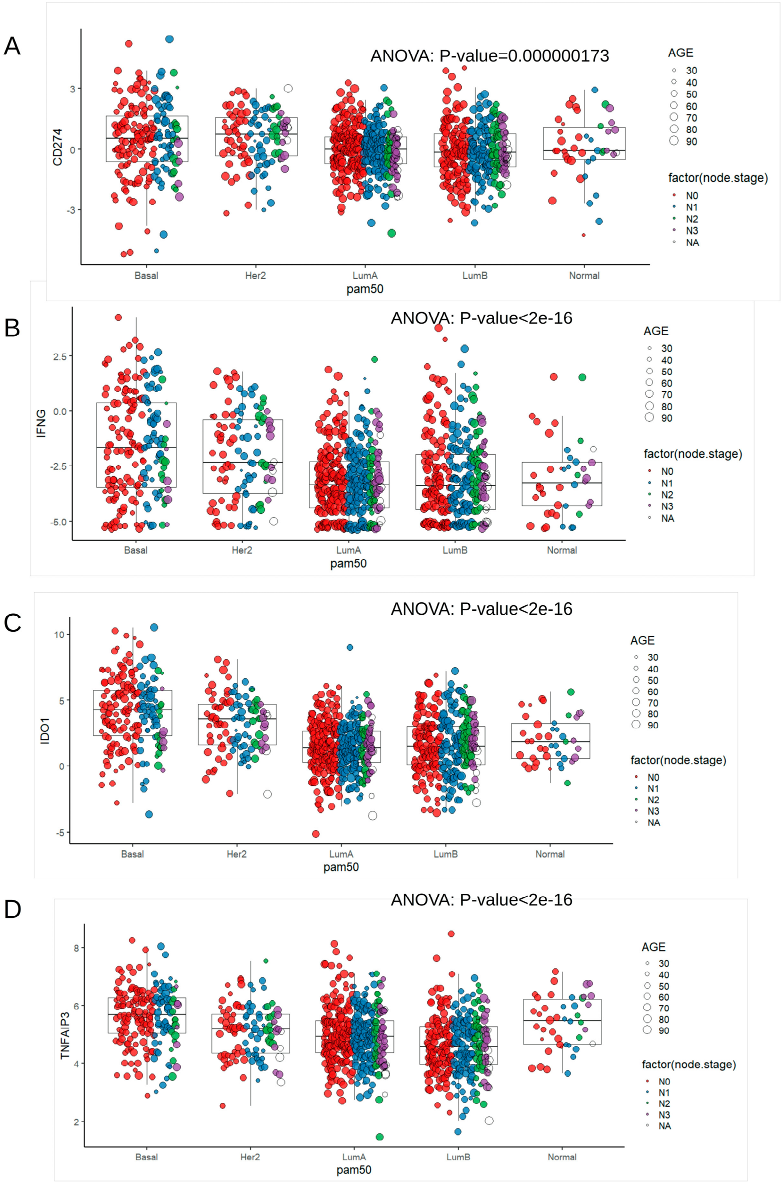

Figure S3. Breast tumor expression of markers included in CD274-ferrptosis score (TCGA cohort): (A) CD274 expression stratified on pam50 molecular classification (dot colors represent node stages and dot size is relative to the age of diagnosis); (B) IFNG expression stratified on pam50 molecular classification (dot colors represent node stages and dot size is relative to the age of diagnosis); (C) IDO1 expression stratified on pam50 molecular classification (dot colors represent node stages and dot size is relative to the age of diagnosis); (D) TNFAIP3 expression stratified on pam50 molecular classification (dot colors represent node stages and dot size is relative to the age of diagnosis).

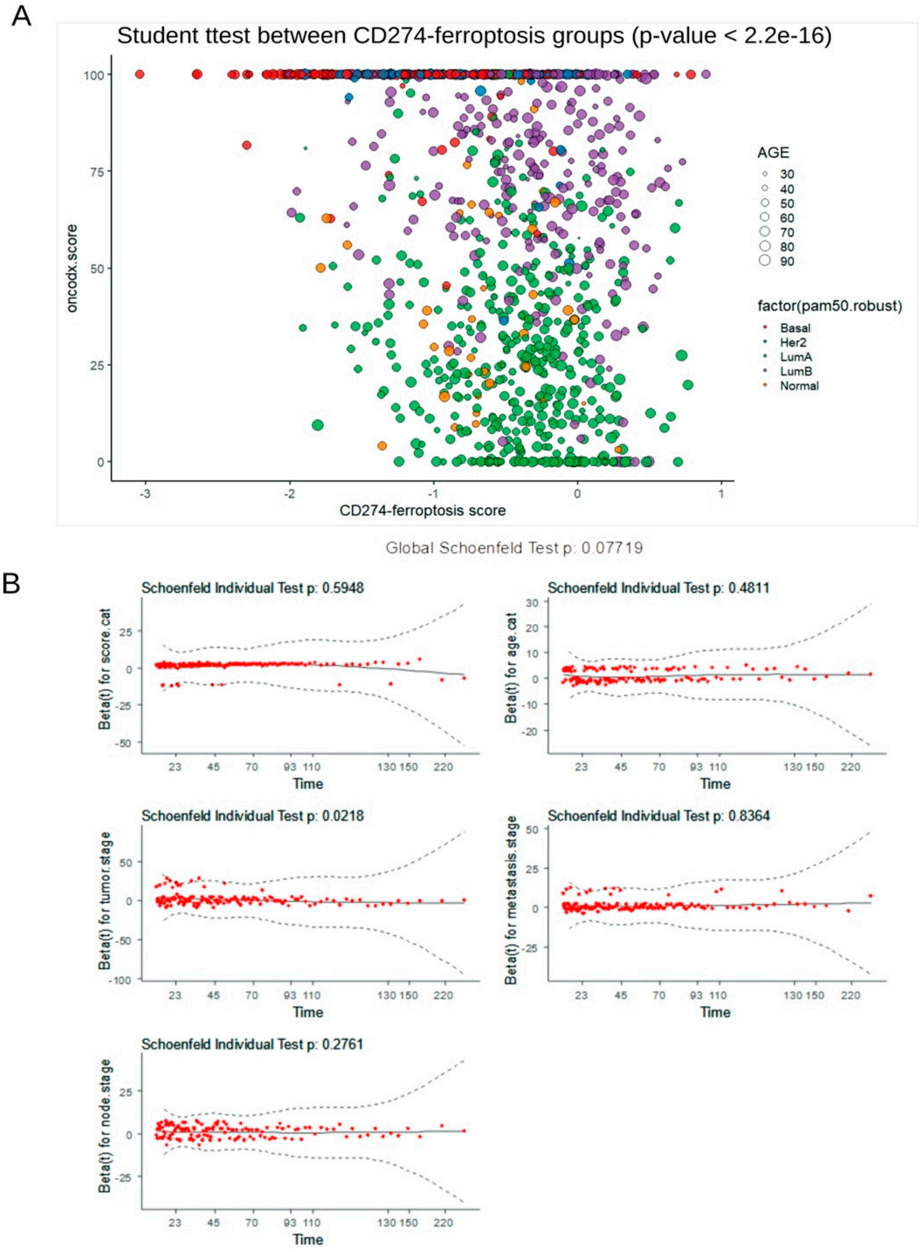

Figure S4. OncotypeDx quantification in breast tumor sample and Schoenfeld test for multivariable survival model (TCGA cohort): (A)Scatterplot of oncoDx score versus CD274-ferroptosis driver score stratified on pam50 molecular classification (Student ttest on OncoDx score was done between CD274-ferroptosis groups low and high); (B) Schoenfeld test done on covariates included in overall survival multivariable model of the TCGA cohort.

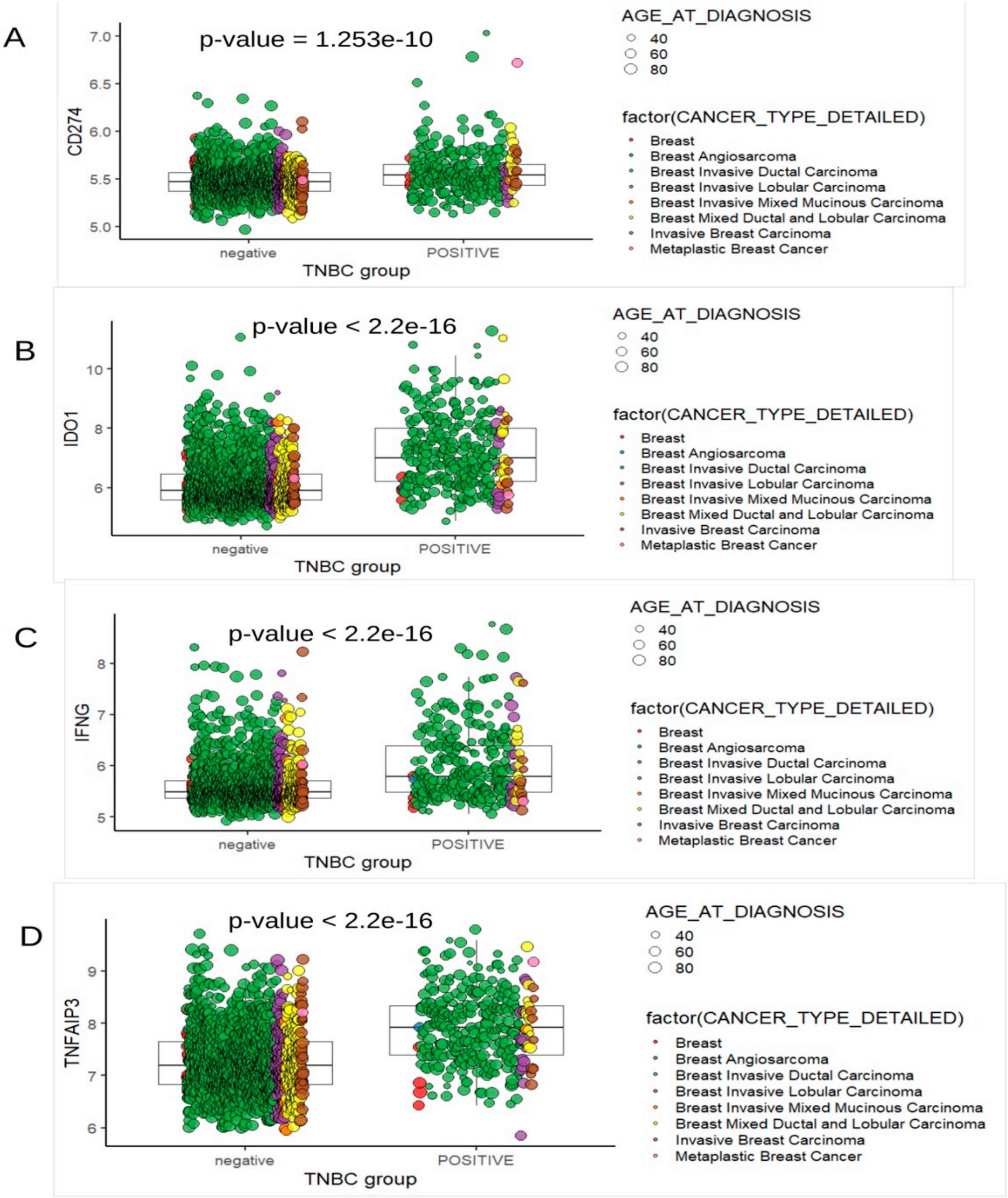

Figure S5. Overexpression of CD274, IDO1, IFNG and TNFAIP3 in TNBC subgroup of METABRIC cohort: (A) Boxplot of CD274 expression stratified on TNBC subgroup membership; (B) Boxplot of IDO1 expression stratified on TNBC subgroup membership; (C) Boxplot of IFNG expression stratified on TNBC subgroup membership; (D) Boxplot of TNFAIP3 expression stratified on TNBC subgroup membership.
